# SG-APSIC1101: Virulence factors and antimicrobial resistance in coagulase-negative staphylococci isolated from blood of neonates

**DOI:** 10.1017/ash.2023.6

**Published:** 2023-03-16

**Authors:** Svetlana Kolesnichenko, Irina Kadyrova, Alyona Lavrinenko, Lyudmila Akhmaltdinova, Lyudmila Panibratec

**Affiliations:** Karaganda Medical University, Kazakhstan; Karaganda, Karaganda Medical University, Kazakhstan; Karaganda, Karaganda Medical University, Kazakhstan; Karaganda Medical University, Kazakhstan; Karaganda, Regional Clinical Hospital of Karaganda, Perinatal Center No. 2, Kazakhstan

## Abstract

**Objectives:** To determine virulence genes and sensitivity to antibacterial drugs of *Staphylococcus epidermidis* isolated from blood cultures of newborns. **Methods:** A study of сoagulase-negative *Staphylococcus* (CoNS) from newborns with sepsis was conducted in the regional perinatal center in Karaganda, Kazakhstan. Blood-culture identification was performed using MALDI-TOF MS. Virulence factors were determined on primers (*sdrG*, *sdrG*, *atl*, *lip*, *nuc*, *ebh*, *hlb*, *sspA*, *sspB*, and *gehD*) with PCR (Bio-Rad CFX 96). Susceptibility to antibiotics determination was carried out using the disc-diffusion method. Testing with cefoxitin was used to detect methicillin resistance in staphylococci. **Results:** Overall, 18 *Staphylococcus epidermidis* isolates from blood cultures of newborns with sepsis were investigated from January to December 2021. The frequency of detection of virulence genes was distributed as follows: *atl* (94.5%), *sspB* (94.5%), *sspA* (89%), *gehD* (89%), *ebh* (89%), *hlb* (72%), *sdrG* (39%), *sdrF* (28%), *nuc* (28%), and *lip* (13%). Also, 10 isolates (55%) were resistant to cefoxitin (MRSE). Furthermore, 72% of *S. epidermidis* isolates showed resistance to azithromycin and 33% were resistant to clindamycin and gentamicin. Also, 39% of strains were resistant to fluorchinolones. All isolates were susceptible to vancomycin, linezolid, and fusidic acid. **Conclusions:** *S. epidermidis* strains isolated from blood cultures had high rates of exoenzymes sspB, sspA, gehD, autolysin (atl), β-hemolysin (hlb), and cell-wall–associated fibronectin-binding protein (ebh). Among 18 neonatal sepsis pathogens, 10 (55%) were MRSE, so it is necessary to pay attention to antibiotic therapy adjustment.

